# Successful resection of a large asymptomatic lipoma in the left ventricle with infiltration of the myocardial free wall: a case report

**DOI:** 10.1093/ehjcr/ytaf395

**Published:** 2025-08-09

**Authors:** Tobias Heer, Christian Hagl, Stefan Sack

**Affiliations:** Department of Cardiology, Academic Teaching Hospital of LMU Klinikum, München Klinik Neuperlach, Oskar-Maria-Graf-Ring 51, 81373 Munich, Germany; Department of Cardiac Surgery, LMU Klinikum, Marchioninistr. 15, 81377 Munich, Germany; DZHK Partner Site Munich Heart Alliance, LMU Klinikum, Marchioninistr. 15, 81377 Munich, Germany; Department of Cardiology, Academic Teaching Hospital of LMU Klinikum, München Klinik Neuperlach, Oskar-Maria-Graf-Ring 51, 81373 Munich, Germany

A 45-year-old woman was referred for ECG prior to a routine knee operation. Her cardiac history was unremarkable, and she had never experienced any cardiac symptoms. The ECG revealed T wave inversions in leads I, II, III, aVF, and V4–V6, prompting an echocardiography. This revealed a large mobile structure in the left ventricle, leading to further evaluation with cardiac magnetic resonance (CMR) imaging: (*Panel A*) SSFP sequences, three-chamber view, confirming a large tumour (3 × 3 × 6 cm) infiltrating the inferolateral wall of the left ventricle, accompanied by myocardial thinning and involvement of the mitral subvalvular apparatus. In the native T1 sequence, the tumour appeared hyperintense (*Panel B*), whereas in the T1 sequence with fat suppression, it was hypointense (*Panel C*). Based on these findings, the CMR diagnosis was lipoma.

**Figure:**
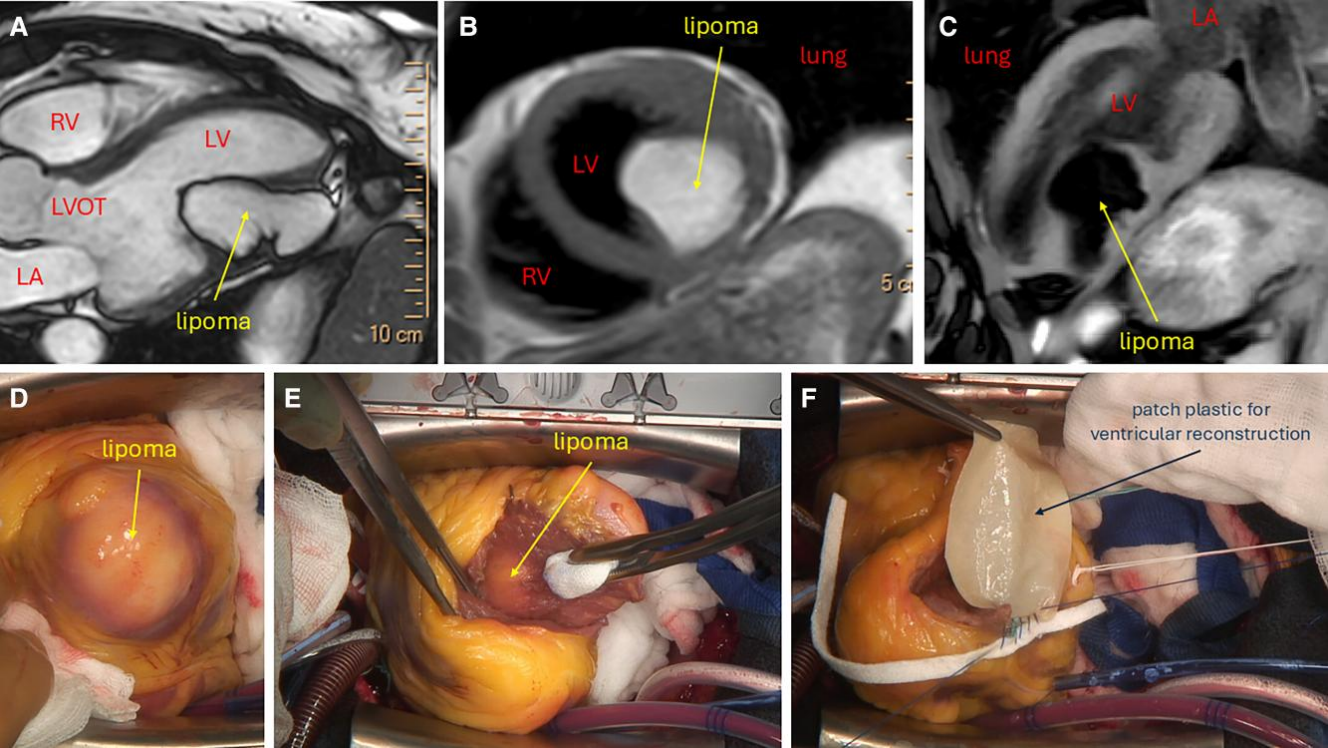


The case was discussed in the heart team and we decided to perform a surgical resection for the following reasons: (i) the extensive infiltration of the myocardium with thinning of the free wall and with involvement of the mitral subvalvular apparatus (heart transplantation was the ultimate solution in other published cases), (ii) to prevent overgrowth of the already very large lipoma in the left ventricle with potential impairment of left ventricular filling and further development of heart failure with increasing volume, (iii) to prevent thromboembolic complications, and (iv) because of the young age of our patient. *Panels D–F* show intraoperative images: view of the heart after sternotomy (*Panel D*), the lipoma inside the heart (*Panel E*), and the patch plastic for ventricular reconstruction (*Panel F*). Histological examination confirmed the diagnosis of cardiac lipoma which are rare primary tumours, are usually asymptomatic, and carry a good prognosis. CMR is the confirmatory investigation of choice. The treatment is individualized.

## Data Availability

All data are incorporated into the article.

